# SPINA Carb: a simple mathematical model supporting fast in-vivo estimation of insulin sensitivity and beta cell function

**DOI:** 10.1038/s41598-022-22531-3

**Published:** 2022-10-21

**Authors:** Johannes W. Dietrich, Riddhi Dasgupta, Shajith Anoop, Felix Jebasingh, Mathews E. Kurian, Mercy Inbakumari, Bernhard O. Boehm, Nihal Thomas

**Affiliations:** 1 Diabetes, Endocrinology and Metabolism Section, Department of Internal Medicine I, St. Josef Hospital, Ruhr University Bochum, NRW, Gudrunstr. 56, 44791 Bochum, Germany; 2 Diabetes Centre Bochum-Hattingen, St. Elisabeth-Hospital Blankenstein, Im Vogelsang 5-11, 45527 Hattingen, NRW Germany; 3 Centre for Rare Endocrine Diseases, Ruhr Centre for Rare Diseases (CeSER), Ruhr University Bochum and Witten/Herdecke University, Alexandrinenstr. 5, 44791 Bochum, NRW Germany; 4 Centre for Diabetes Technology, Catholic Hospitals Bochum, Gudrunstr. 56, 44791 Bochum, NRW, Germany; 5 Department of Endocrinology, Diabetes and Metabolism, Christian Medical College, Vellore, 632004 India; 6 Lee Kong Chian School of Medicine, Nanyang Technological University Singapore, 11 Mandalay Road, Singapore, 308232 Singapore; 7 Department of Internal Medicine I, Ulm University Medical Centre, Ulm University, 89070 Ulm, Germany; 8 Department of Endocrinology, Tan Tock Seng Hospital, Singapore, Singapore

**Keywords:** Diabetes, Control theory

## Abstract

Modelling insulin-glucose homeostasis may provide novel functional insights. In particular, simple models are clinically useful if they yield diagnostic methods. Examples include the homeostasis model assessment (HOMA) and the quantitative insulin sensitivity check index (QUICKI). However, limitations of these approaches have been criticised. Moreover, recent advances in physiological and biochemical research prompt further refinement in this area. We have developed a nonlinear model based on fundamental physiological motifs, including saturation kinetics, non-competitive inhibition, and pharmacokinetics. This model explains the evolution of insulin and glucose concentrations from perturbation to steady-state. Additionally, it lays the foundation of a structure parameter inference approach (SPINA), providing novel biomarkers of carbohydrate homeostasis, namely the secretory capacity of beta-cells (SPINA-GBeta) and insulin receptor gain (SPINA-GR). These markers correlate with central parameters of glucose metabolism, including average glucose infusion rate in hyperinsulinemic glucose clamp studies, response to oral glucose tolerance testing and HbA1c. Moreover, they mirror multiple measures of body composition. Compared to normal controls, SPINA-GR is significantly reduced in subjects with diabetes and prediabetes. The new model explains important physiological phenomena of insulin-glucose homeostasis. Clinical validation suggests that it may provide an efficient biomarker panel for screening purposes and clinical research.

## Introduction

Mathematical modelling, particularly when combined with computer simulation, is increasingly favoured in the field of diabetes research^1,2^. Some of the published models may also be used for medical decision making and other diagnostic purposes^3,4^, or for prognostic assessment^5^. For example, this applies to the homeostasis model assessment/insulin resistance assessment/ß-cell function (HOMA-IR and HOMA-Beta)^6^ and the quantitative insulin sensitivity check index (QUICKI)^7^. More recently, models of the insulin-glucose feedback loop have been increasingly applied for therapeutic purposes as well, particularly in the context of sensor-augmented and closed-loop insulin delivery systems^8–11^. The growing success in clinical applications of modelling are mainly a result of both advanced insights into the physiology of insulin-glucose homeostasis, the availability of continuous glucose measurement technologies and improved methods of numeric simulation^12^.

However, modelling usually represents a necessary compromise between the two often opposing goals of simplicity and accurate reproducibility. The question frequently arises as to where to draw the line between simplification and comprehensive coverage of physiological phenomena, and the location of this line may depend on the specific requirements of the application. Therefore, the classical models that underlie the methods of calculating HOMA and QUICKI provide an, although in some aspects over-simplified, reasonably pragmatic approach, since they facilitated the development of rapid and economically efficient diagnostic index methods^13^. On the other hand, complex approaches such as the Sorensen model and its derivations are useful for comprehensive understanding of detailed causal networks^14,15^, although they are less applicable for routine clinical purposes. Yet, the consistency and predictive power of the HOMA methodology is limited^16^. This applies to both assessment of insulin resistance^17–19^ and estimation of beta-cell function^20,21^.

With the advent of novel nonlinear methods for modelling and simulating feedback loops, and owing to the continued evolution of physiological knowledge and metabolic pathways it is time to revisit the methodology of screening for both insulin resistance and beta-cell function.

For this purpose, we ventured to develop a novel model for insulin-glucose homeostasis, which amalgamates the targets of simplicity and accuracy. This model is primarily based on pharmacokinetic data and fundamental physiological principles including non-competitive inhibition and saturation kinetics, as they are described by the Monod equation and applied in Michaelis–Menten kinetics, receptor theory and the Langmuir adsorption model.

Our main goals were to provide a simple and physiologically comprehensible model, to enable “vertical translation” from the molecular to a whole-body scale and to provide the foundation of a cost-effective diagnostic procedure requiring not more than a single fasting measurement of insulin and glucose. In favour of this goal of simplification, some factors such as glucagon production and the impact of incretins were deliberately omitted.

The diagnostic procedure derived from the model was designed to deliver information on insulin sensitivity and beta cell function, which are represented in the main building blocks of the theory and have been established as pathophysiological substrate for the development of diabetes and its classification.

## Methods

The methodology comprises four parts, (1) the development of a concise nonlinear mathematical model of insulin-glucose homeostasis, (2) the implementation of this model through computer simulation, (3) the derivation of static function tests for insulin sensitivity and beta-cell function, and (4) the validation of these structural parameters in ethnically different clinical cohorts.

### Mathematical model

**Figure 1:**
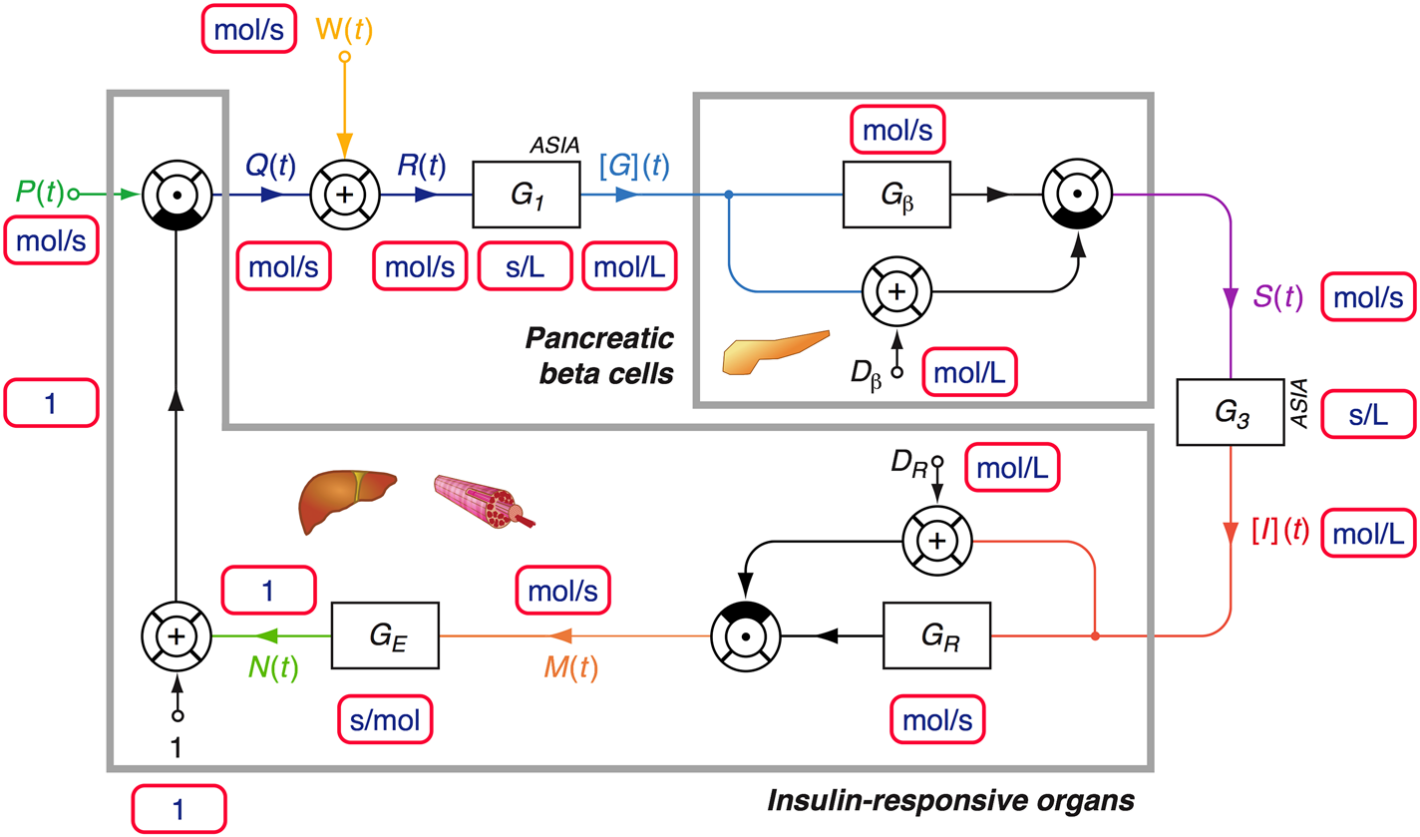
Information processing structure of the feedback model. [G](t): glucose concentration; S(t): insulin secretion rate; [I](t): insulin concentration; M(t): proximal insulin signalling; N(t) distal insulin signalling; P(t): constitutive glucose production rate; Q(t): regulated glucose production rate; W(t): intestinal glucose absorption rate; R(t): glucose arrival rate. G_1_ and G_3_ represent gains of ASIA elements (see text for their derivation). Units of measurement are displayed in the small rounded boxes with red margins.

The subsequently described model is based on the nonlinear MiMe-NoCoDI platform for endocrine feedback loops^22^. This platform has, however with a different model structure, previously been successfully implemented for several endocrine control motifs including thyroid homeostasis and hypothalamus–pituitary–adrenal (HPA) axis^23,24^. It combines saturation kinetics (in the form of Michaelis–Menten, Monod or Langmuir equations, for stimulating pathways) and non-competitive inhibition (for inhibiting relations). Pharmacokinetic properties of distribution and elimination are modelled as first-order kinetics. For the purpose of modelling insulin-glucose homeostasis, the platform has been adapted to account for the specific causal interactions in this feedback control system on the basis of the available experimental evidence (Fig. 1). The supplementary material S1 can be accessed for a full mathematical exposition and dimensional analysis.

The Rates of secretion or absorption are translated to concentrations by fundamental pharmacological principles of elimination and distribution. This process is modelled with so-called ASIA (Analog Signal memory with Intrinsic Adjustment) elements implementing first-order kinetics in the time domain^22^. It can be expressed in form of the *von Bertalanffy* growth function, where the change rate of the concentration substance *y* depends on a controlling input signal *x*(*t*), the volume of distribution *V*_*D*_ and a clearance exponent (rate constant) *β* with

1 $$\frac{dy}{dt}=\frac{x\left(t\right)}{{V}_{D}}-\beta y\left(t\right).$$

In steady state ($t\to \infty$), the concentration of *y* results as

2 $${[y]}_{\infty }=\frac{\alpha {x}_{\infty }}{\beta },$$

where the input signal *x*_*∞*_ is assumed to be constant and *α* = 1/*V*_*D*_. The rate constant depends on

3 $$\beta = \frac{{{\text{ln}}\left( 2 \right)}}{{t_{1/2} }}$$

with regards to the half-life of a respective substance. The transitional behaviour is described as

4 $$\left[ y \right]\left( t \right) = \frac{\alpha x\left( t \right)}{\beta } + Ke^{{{-}\beta t}} .$$

Of note, in steady state, the behaviour of an ASIA element can be simplified as a linear factor *G* = *α*/*β*, so that

5 $${[y]}_{\infty }=G{x}_{\infty }$$

Therefore, we can define the ASIA element for glucose concentration as

6 $${G}_{1}=\frac{{\alpha }_{G}}{{\beta }_{G}} .$$

Under this assumption the plasma glucose concentration *G*(*t*) can be approximated with

7 $$G\left(t\right)={G}_{1}R(t)$$

as a linear function of the glucose arrival rate *R*(*t*), which is defined as the sum of the regulated endogenous glucose production rate *Q*(*t*) and the intestinal glucose absorption rate *W*(*t*), so that Eq. (7) can be expressed in the form

8 $$G\left(t\right)={G}_{1}\left[W\left(t\right)+Q(t)\right]$$

as a linear function of glucose production and absorption.

Although secondary insulin signalling *N*(*t*) is fed in via a non-competitive inhibiting mechanism

9 $$Q\left(t\right)=\frac{P\left(t\right)]}{1+N(t)}$$

the insulin concentration itself undergoes saturation kinetics, which is expressed by the term

10 $$N\left(t\right)=\frac{{G}_{E}{G}_{R}I(t)}{{D}_{R}+I(t)}.$$

In summary, insulin concentration results as the weighted sum of *W*(*t*) and a non-competitive mechanism comprising basal glucose production *P*(*t*), insulin concentration *I*(*t*) and several constant structure parameters of the feedback loop (Table 1):

**Table 1:** Parameters used for predicting the steady state and for computer simulations.

Parameter	Explanation	Value	Source or references
*α*_*G*_	Dilution factor (1/*V*_*D*_) for glucose	0.11 L^−1^	^32,53^
*β*_*G*_	Clearance exponent (rate constant) for glucose	7.1e–4 s^−1^	^32,54^
*G*_1_	*α*_*G*_/*β*_*G*_		by definition
*G*_*β*_	Secretory capacity of beta cells	2.8 pmol/s	Estimated from NHANES data
*D*_*β*_	EC_50_ of glucose at beta cells	7 mmol/L	^55,56^
*α*_*I*_	Dilution factor (1/*V*_*D*_) for insulin	0.2 L^−1^	^57^
*β*_I_	Clearance exponent for insulin	3.4e–3 s^−1^	^58,59^
*G*_3_	*α*_*I*_/*β*_*I*_		by definition
*G*_*R*_	Insulin receptor gain	2.3 mol/s	Estimated from NHANES data
*D*_*R*_	EC_50_ of insulin at its receptor	1.6 nmol/L	^60^
*G*_*E*_	Effector gain	50 s/mol	Calibration factor
*P*	Constitutive endogenous glucose production	150 µmol/s	Derived from^31,32^
*W*	Intestinal glucose absorption rate	0 µmol/s	by definition

11 $$G\left(t\right)={G}_{1}W\left(t\right)+\frac{{G}_{1}P\left(t\right)]}{1+\frac{{G}_{E}{G}_{R}I(t)}{{D}_{R}+I(t)}}$$

Similar to Eq. (6), the ASIA element for insulin concentration is defined as

12 $${G}_{3}=\frac{{\alpha }_{I}}{{\beta }_{I}} .$$

Therefore, insulin concentration depends on glucose concentration and constant parameters with

13 $$I\left(t\right)=\frac{{G}_{3}{G}_{\beta }G(t)}{{D}_{\beta }+G(t)}.$$

With

14 $${K}_{1}=\frac{{G}_{E}{G}_{R}{G}_{3}{G}_{\beta }}{{D}_{R}+{G}_{3}{G}_{\beta }}$$

and

15 $${K}_{2}=\frac{{D}_{R}{D}_{\beta }}{{D}_{R}+{G}_{3}{G}_{\beta }}$$

the glucose concentration can be expressed in an iterative equation as

16 $$G\left(t+1\right)={G}_{1}W\left(t\right)+\frac{{G}_{1}P\left(t\right)}{1+\frac{{K}_{1}G(t)}{{K}_{2}+G(t)}}$$

being dependent on *P*(*t*), *W*(*t*) and structure parameters only.

For the fasting steady-state (equifinal) case with *W*(*t*) = 0 and $t\to \infty$, the iterative Eq. (16) can be rewritten with $a=1+{K}_{1}$, $b={K}_{2}-{G}_{1}P(\infty )$ and $c=-{G}_{1}{K}_{2}P(\infty )$ as the quadratic equation

17 $$a{G(\infty )}^{2}+bG\left(\infty \right)+c=0.$$

Since $b<{b}^{2}-4ac>0$ Eq. (17) has the two solutions

18 $${G\left(\infty \right)}_{\mathrm{1,2}}=\frac{-b\pm \sqrt{{b}^{2}-4ac}}{2a}.$$

The positive solution represents the equifinal glucose concentration $G\left(\infty \right)$ in the steady fasting state.

### Computer simulations

In order to study the temporal behaviour of the feedback loop, the model has been implemented with a computer simulation (SimulaBeta)^26^. The software has been written in Object Pascal with the Lazarus IDE (version 2.0.10)^27^ for Free Pascal (version 3.0.4)^28^ and utilises the class library CyberUnits Bricks^29^ for simulations in life sciences (version 1.1.1). Additional code for some visualisation purposes has been written in the language S with the R environment for statistical computation (version 3.6.3)^30^. The parameters used for simulation and steady-state prediction have been reported in Table 1. For simulation, the constitutive glucose production rate *P*_0_ was defined to be 150 µmol/s, so that it delivers a fasting regulated glucose arrival rate (*R*) between 10 and 100 µmol/s^31,32^ (see supplementary information S1 for a detailed explanation of its derivation).

### Derivation of a structure parameter inference approach (SPINA) as static function test

Under the assumption of steady-state fasting conditions, the equations describing the behaviour of the feedback loop can be solved for constant structure parameters. Here, we were interested in the gains of beta cells (*G*_*β*_) and insulin sensitivity (*G*_*R*_).

From the model equations *G*_*β*_ can be estimated as

19 $${\widehat{G}}_{\beta }=\frac{[I](\infty )({D}_{\beta }+\left[G\right](\infty ))}{{G}_{3}[G](\infty )}$$

and *G*_*R*_ as

20 $${\widehat{G}}_{R}=\frac{{G}_{1}P(\infty )({D}_{R}+\left[I\right](\infty ))}{{G}_{E}\left[I\right](\infty )[G](\infty )}-\frac{{D}_{R}}{{G}_{E}[I](\infty )}-\frac{1}{{G}_{E}}$$

For a simplified notation, we will subsequently refer to ${\widehat{G}}_{\beta }$ as SPINA-GBeta and to ${\widehat{G}}_{R}$ as SPINA-GR, consistent with the notation used for model-based assessment of thyroid homeostasis^33^.

### Validation studies

The validity and diagnostic utility of the newly defined structure parameters were evaluated in two independent cohorts from the USA and India. For this purpose, we re-analysed anthropometric data and results of oral glucose tolerance testing from the NHANES 2007/2008 cohort for a potential correlation to SPINA-GBeta and SPINA-GR^34^. Additionally, we calculated the same parameters in an independent cohort from rural India, based on observations from hyperinsulinemic-euglycemic clamp (HEC) studies in a homogenous cohort of Asian Indian males with low body mass index^35^. Results for SPINA-GBeta and SPINA-GR were compared with surrogate indices, namely HOMA-Beta, HOMA-IR and QUICKI. All biomarkers of insulin-glucose homeostasis were calculated from fasting glucose and insulin concentrations. In order to obtain information on re-test reliability and intra-individual clustering repeated measurements of fasting insulin and glucose concentrations were performed with an interval of four days between repeats.

The inclusion criteria for the re-analysis of the NHANES cohort were the availability of data on oral glucose tolerance testing (OGTT) and the presence of somatometric measurements (body mass and length) as well as fasting glucose and insulin concentrations. Exclusion criteria were treatment with insulin or oral antidiabetic agents. The second validation was done from data from HEC studies in non-obese (BMI < 20 kg/m^2^), young, normoglycemic Asian Indian males from Southern India. The protocol for the comprehensive study was approved by the Institutional Review Board (IRB) of Christian Medical College, Vellore, India (Research Committee Minute Number: 5879, 2006 and Administrative Committee Minute Number: 50-y: 6-2006) and was performed in accordance with the Declaration of Helsinki. This study included males aged between 18 and 22 years who were normoglycemic, normolipidemic individuals with no history of smoking or alcoholism, hepatic diseases, HIV, malignancies of any form and drug overuse. Informed consent was obtained from all subjects, prior to inclusion in the study. The participants underwent anthropometry and whole-body composition analysis by Dual Energy X-ray absorptiometry (DXA). The DEXA is a non-invasive, gold standard technique that uses a dual energy X ray beam to quantify soft tissues and bones. Measures of body composition, namely fat mass, lean mass and fat-free mass were quantified by a DEXA scanner (Hologic DEXA Discovery QDR 4500). The scanner was calibrated daily using an aluminum phantom. Bilateral sections and whole body composition data were obtained by analysis of the regions of interest (ROI) using APEX software^36^. All subjects included in the HEC study were normoglycemic and healthy. They were not on any medications or supplements that could interfere with the results of insulin or glucose determinations, or the clamp procedures.

### Hyperinsulinemic Euglycemic Clamp (HEC) methodology used in the study

Participants of the study were apprised on the clamp procedure. The eligible study participants reported to the metabolic study centre at 07:00 h after an overnight fast (lasting at least 8 h after dinner) and no consumption of any form of beverages in the morning. A physician examined the vital physiological parameters, prior to the start of the hyperinsulinemic euglycemic clamp (HEC) procedure. All participants underwent a non-tracer based 120-min HEC procedure for assessment of whole-body insulin sensitivity. In the HEC procedure, two indwelling intravenous catheters were inserted contralaterally in the veins of the antecubital fossa. In one catheter, a continuous insulin infusion was initiated, and the flow rate was maintained at 40 mU/kg/min using an automated infusion pump (Terumo infusion pump TE-112) during the entire duration of the clamp. To maintain euglycemia (5 mmol/L), 25% dextrose solution was infused with meticulously adjusted flow rates. The dextrose infusion rate was adjusted to maintain a stable plasma glucose concentration of 5 mmol/L throughout the clamp procedure. Plasma glucose levels were measured by drawing blood samples from the other antecubital vein, every 5 min, using a bedside glucose analyser (Analox GM-9D). Blood samples for biochemical estimation of insulin, C-peptide and plasma glucose were drawn at baseline and at the end of the steady state phase (i.e., last 30 min of the basal phase and the last 30 min of the clamp period)^35^. The measure of whole-body insulin sensitivity was designated as glucose disposal rate (M-value) and was derived during a steady state wherein euglycemia (5 mmol/L of plasma glucose) was achieved by infusing high levels of insulin during the 2-h HEC procedure. The steady-state was calculated between 60 and 120 min after the start of the insulin infusion, based on the formula of DeFronzo et al.^37^.

### Laboratory procedures

In the group undergoing HEC plasma glucose levels were measured by the glucose-oxidase method. Serum insulin and C-peptide levels were measured by the chemiluminescence method using diagnostic kits supplied by Siemens, on the Immulite 2000 system (Siemens Healthcare Diagnostic Products, Llanberis, Gwynedd, UK). Chemistry and Immunoassay controls supplied by Bio-Rad were used as internal precision controls (coefficient of variation (CV) 10.2% for insulin and 3.7% for C-peptide)^35^.

In the NHANES cohort fasting glucose concentration was measured with UniCel® DxC800 Synchron and Synchron LX20 assays (Beckman Coulter, Inc, Brea, Ca., USA) with an analytical range of 0.16–33.3 mmol/L and up to 66.7 mmol/l with ORDAC (overrange detection and correction). Serum insulin concentrations were determined with an ELISA kit (Mercodia AB, Uppsala, Sweden). The detection limit was 6 pmol/L. Whole-blood glycohemoglobin (HbA1c) concentrations were measured with an A1c 2.2 Plus or an A1c G7 HPLC Glycohemoglobin Analyzer (both Tosoh Medics, Inc., San Francisco, Ca., USA). The reportable range was between 3.4% and 18.8% or 3.0% and 19.0%, respectively.

### Statistical methods

The Statistical analyses performed with custom S scripts written for the environment R 3.6.3 on macOS^30^. Biomarkers in different groups (no diabetes, prediabetes and diabetes) were compared via Kruskal–Wallis tests and post-hoc pairwise Wilcoxon-Mann–Whitney *U* test. Alpha error correction for multiple testing was performed with the Benjamini–Hochberg procedure. Correlations between continuous variables were evaluated with Spearman’s rank correlation, and Zou’s procedure was used to obtain confidence intervals for comparing correlations for new and traditional parameters^38^. Bland–Altman plots were used to test the agreement between traditional and novel calculated parameters. Ergodicity of biomarkers was evaluated as repeatability from intraindividual and interindividual variances with

21 $$e=\frac{{Var}_{interindividual}}{{Var}_{intraindividual}+{Var}_{interindividual}}.$$

In ergodic systems intraindividual statistical moments are equal or at least very similar to parameters of interindividual statistics. Intraindividual clustering implies low ergodicity.

### Ethics approval and consent to participate

The NHANES protocol has been approved by the NCHS Research Ethics Review Board (ERB) of the US National Center for Health Statistics (Protocol #2005-06). The protocol for the glucose clamp study was approved by the Institutional Review Board (IRB) of Christian Medical College, Vellore, India (Research Committee Minute Number: 5879, 2006 and Administrative Committee Minute Number: 50-y: 6-2006). All research has been performed in accordance with the Declaration of Helsinki.

## Results

### Modelling and simulation results

With the standard parameters from Table 1, the mathematical model predicts steady-state values for metabolic variables that are located in a physiological range (Supplementary Table S1).

The transitional behaviour of the system in a dynamic simulation is shown for the different initial concentrations in Supplementary Fig. S3. Despite different values at initiation, the same physiological steady-state concentrations are achieved by the feedback loop.

With the parameters of Table 1 the results of simulated oral glucose tolerance tests (oGTT) and frequently sampled intravenous glucose tolerance tests (fsIGT) are within the time-dependent reference ranges of healthy volunteers^39,40^ (Supplementary Table S2 and Supplementary Fig. S4).

### Results of clinical validation

Records of 2472 subjects were selected from the NHANES cohort of 2007–2008, and 100 subjects of a different cohort were included in the clamp study. The basic clinical characteristics of the study populations are reported in Table 2.

**Table 2:** Clinical characteristics of the two studied cohorts.

	NHANES 2007/2008 cohort (n = 2474)	Glucose clamp study in Asian Indian males (n = 100)
Age, years	43.3 ± 20.4	19.8 ± 1.0^†††^
**Sex**		
Female	1242 (50.2%)	0 (0%)
Male	1232 (49.8%)	100 (100%)^†††^
Body surface area, m^2^	1.9 ± 0.3	1.7 ± 0.1^†††^
Body mass index, kg/m^2^	27.5 ± 6.2	19.1 ± 2.8^†††^
Waist circumference, cm	94.6 ± 15.7	69.9 ± 7.4^†††^
Fat-free mass (FFM), kg	N/A	43.4 ± 5.4
Triceps skinfold, mm	18.4 ± 8.3	N/A
Subscapular skinfold, mm	20.0 ± 8.4	N/A
**Diabetes**		
No diabetes	2403 (97.1%)	100 (100%
Prediabetes	31 (1.3%)	0 (0%)
Diabetes	37 (1.5%)	0 (0%)
Fasting glucose (mmol/L)	5.7 ± 1.0	4.8 ± 0.4^†††^
Fasting insulin (pmol/L)	74.8 ± 60.4	24.6 ± 27.1^†††^
HbA1c (%)	5.5 ± 0.6	5.4 ± 0.4
M value (mg/kg/min)	N/A	10.9 ± 3.8

Data are reported as mean ± SD or counts (percentage).

^†††^p < 1e–15 for the comparison of the cohorts.

In the NHANES cohort, HOMA-Beta and SPINA-GR differed between diabetic, prediabetic and nondiabetic subjects (Table 3).

**Table 3:** Biomarkers of insulin-glucose homeostasis in subjects with diabetes, prediabetes and no diabetes of the NHANES cohort and in lean healthy volunteers of the Clamp cohort.

	NHANES cohort			Clamp cohort
	No diabetes (n = 2403)	Prediabetes (n = 31)	Diabetes (n = 37)	Volunteers (n = 100)
HOMA-Beta	122.4 ± 2.2	111.1 ± 12.2	83.1 ± 10.8**	61.7 ± 4.8^†††^
HOMA-IR	3.2 ± 0.1	4.5 ± 0.6	4.2 ± 0.5	0.9 ± 0.1^†††^
QUICKI	0.34 ± 0.00	0.32 ± 0.01	0.33 ± 0.01	0.42 ± 0.01^†††^
SPINA-GBeta (pmol/s)	2.83 ± 0.05	3.29 ± 0.37	2.53 ± 0.31	1.01 ± 0.10^†††^
SPINA-GR (mol/s)	2.34 ± 0.04	1.57 ± 0.19*	1.86 ± 0.30*	8.77 ± 0.64^†††^

Shown are means ± SEM. p * < 0.05, ** < 0.01 compared to subjects without diabetes (comparisons for NHANES cohort only). ^†††^p < 1e–15 for the comparison of the cohorts.

**Figure 2:**
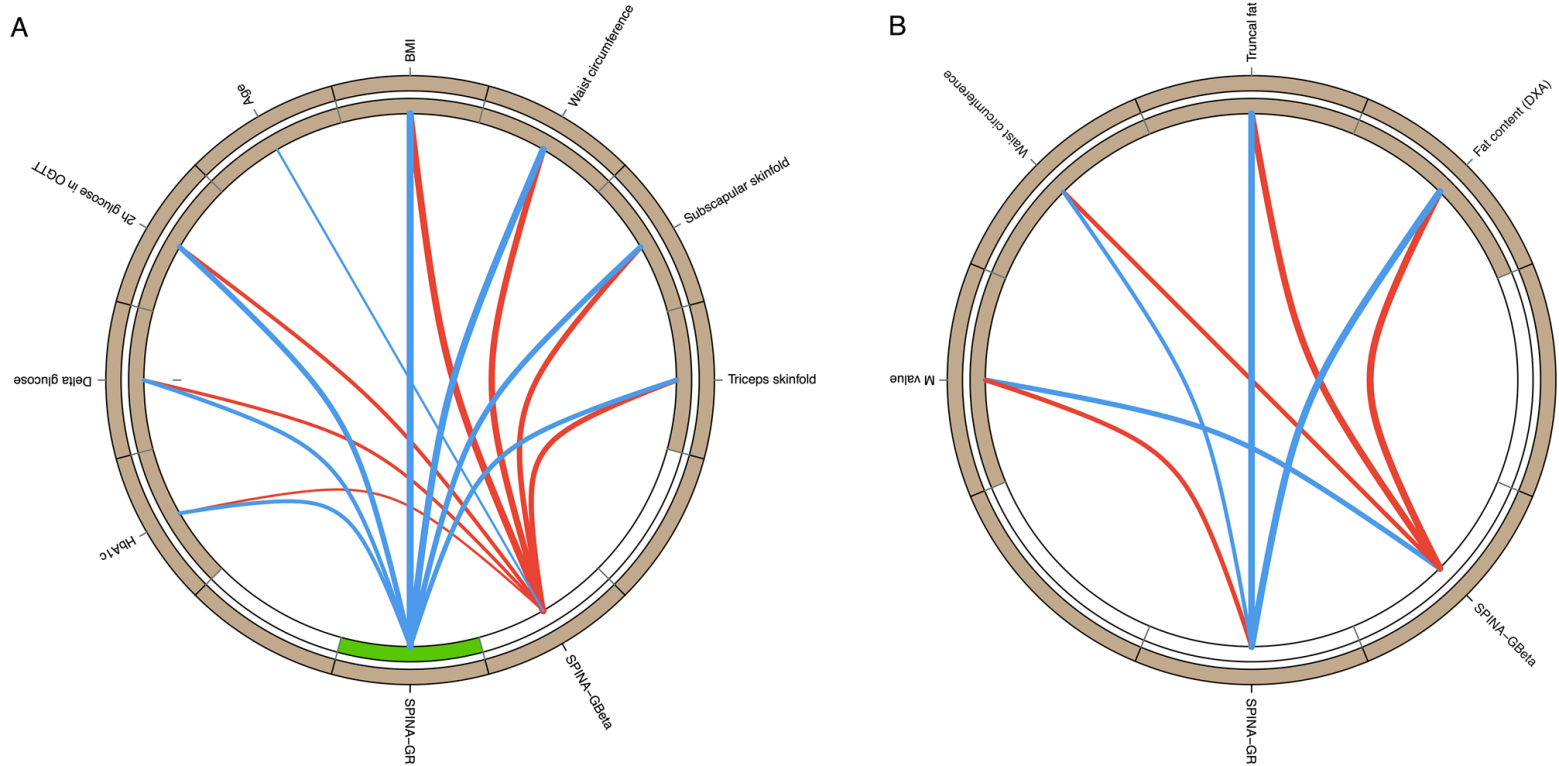
Circular Maps of the correlation networks of novel biomarkers for insulin-glucose homeostasis with anthropometric and experimental data in the NHANES cohort (**A**) and the clamp study (**B**). Shown are significant correlations (p < 0.05) only, and line thickness indicates the strength of negative (blue) or positive (red) correlation. A green segment in the inner ring indicates a reduction of the corresponding biomarker in subjects with diabetes.

All calculated parameters correlated to anthropometric data, response to oGTT and HbA1c values in the NHANES cohort (Table 4 and Fig. 2), but most correlations were weakest for HOMA-Beta. SPINA-GR had a stronger correlation to oGTT results and HbA1c than all other parameters. With the exception of the triceps skinfold, SPINA-GBeta correlated more strongly to clinical parameters than the corresponding HOMA-Beta, in particular to the oGTT results.

**Table 4:** Correlations of calculated biomarkers to somatometric data and response to oral glucose tolerance testing (NHANES cohort) or M-value (clamp cohort), respectively.

	HOMA-Beta	HOMA-IR	QUICKI	SPINA-GBeta	SPINA-GR
**NHANES cohort**					
BMI	0.416^†††^	0.516^†††^	− 0.516^†††^	0.500^†††^∆	− 0.513^†††^
Waist circumference	0.339^†††^	0.498^†††^	− 0.498^†††^	0.456^†††^∆	− 0.502^†††^∆◊
Triceps skinfold	0.371^†††^	0.331^†††^	− 0.331^†††^	0.363^†††^	− 0.320^†††^∆◊
Subscapular skinfold	0.320^†††^	0.383^†††^	− 0.383^†††^	0.375^†††^∆	− 0.381^†††^
2 h glucose in oGTT	0.050*	0.347^†††^	− 0.347^†††^	0.235^†††^∆	− 0.368^†††^∆◊
Glucose rise in oGTT	0.091^†^	0.245^†††^	− 0.245^†††^	0.187^†††^∆	− 0.256^†††^∆◊
HbA1c	− 0.063***	0.219^†††^	− 0.219^†††^	0.104^††^∆	− 0.243^†††^∆◊
**Clamp cohort**					
BMI	0.193	0.056	− 0.054	0.089∆	− 0.049
Waist circumference	0.374***	0.274**	− 0.275**	0.295**	− 0.273**
WHR	0.276**	0.245*	− 0.242*	0.248*	− 0.250*
Truncal fat	0.379***	0.412***	− 0.412^†^	0.408^†^	− 0.411^†^
Fat (DXA)	0.409^†^	0.427^†^	− 0.428^†^	0.429^†^	− 0.429^†^
FFM	0.238*	0.078	− 0.077	0.113∆	− 0.071
M value	− 0.361***	− 0.311**	0.314**	− 0.334***	0.305**

Shown are rho values of Spearman’s rank correlation. p * < 0.05, ** < 0.01, *** < 0.001, ^†^ < 1e–4, ^††^ < 1e–6, ^†††^ < 1e–15 for correlations. ∆ Different from correlation for corresponding HOMA parameter; ◊ different from correlation for QUICKI, based on Zou’s confidence intervals.

**Figure 3:**
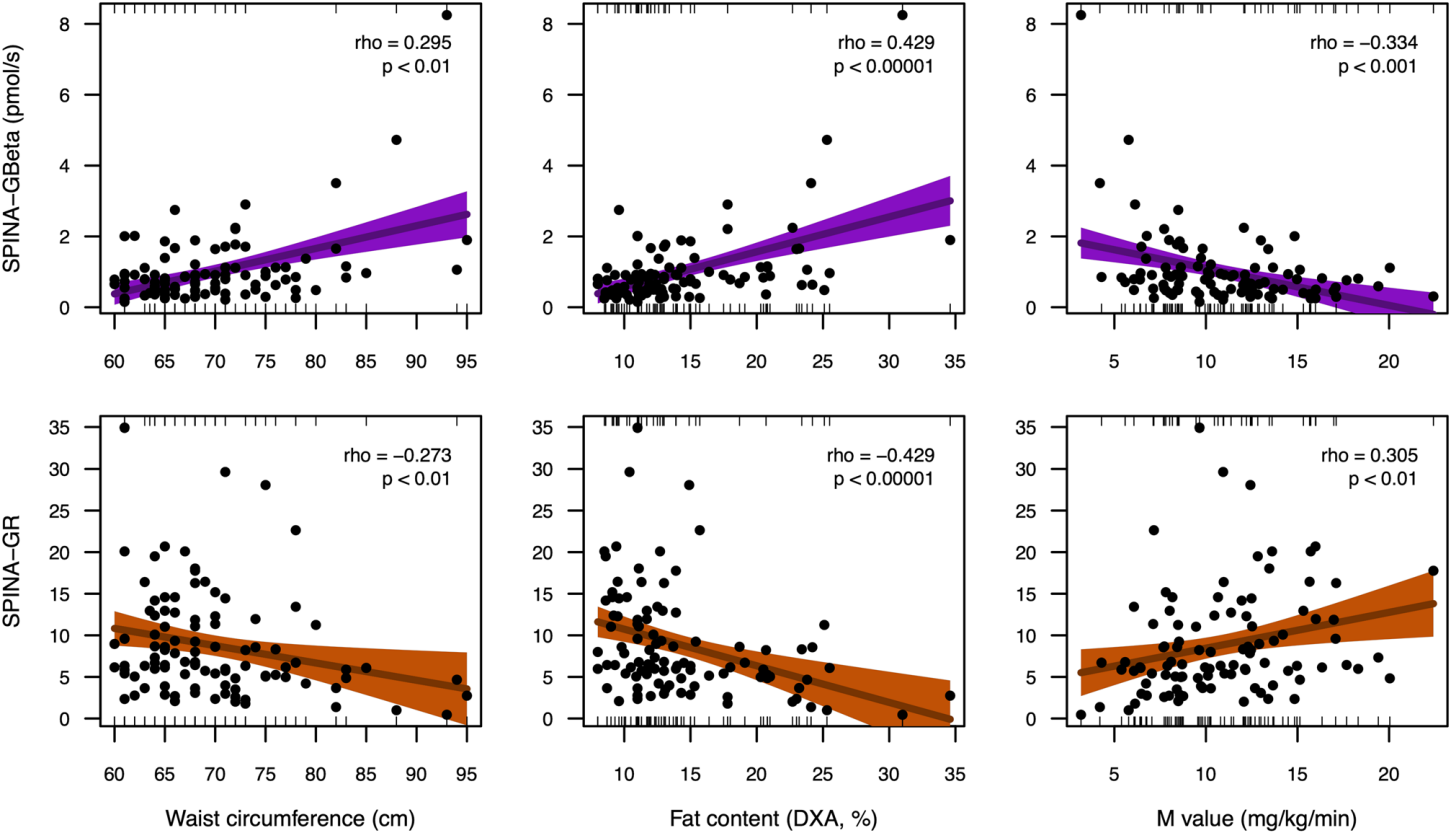
Correlations of SPINA-GBeta and SPINA-GR to waist circumference, fat content and the M value of glucose clamp investigation. p values refer to Spearman’s rho. For greater clarity regression lines of linear models were added together with 95% confidence bands.

Likewise, all parameters showed correlations to the *M* value (measure of whole-body insulin sensitivity) in the glucose clamp experiment, and some of them also correlated to anthropometric measures (Table 4 and Figs. 2 and 3).

As expected, traditional (HOMA and QUICKI) and novel parameters (SPINA) are highly correlated, but Bland–Altman plots reveal proportional bias (supplementary Figs. S5–S7).

### Re-test reliability and ergodicity

All calculated parameters have a comparably high retest reliability, as quantified with Spearman’s *rho* from results in repeated measurements (Supplementary Table S3). Among the biomarkers for beta-cell function, SPINA-GBeta has a higher re-test reliability than HOMA-Beta. Based on Spearman’s *rho*, SPINA-GR is slightly more reliable than the other two markers for insulin sensitivity, although its repeatability quantified by *e* is lower. All biomarkers have low ergodicity as demonstrated by a quite high contribution of interindividual variance to the total variance (Supplementary Table S3).

### Relation between insulin resistance and beta cell function

**Figure 4:**
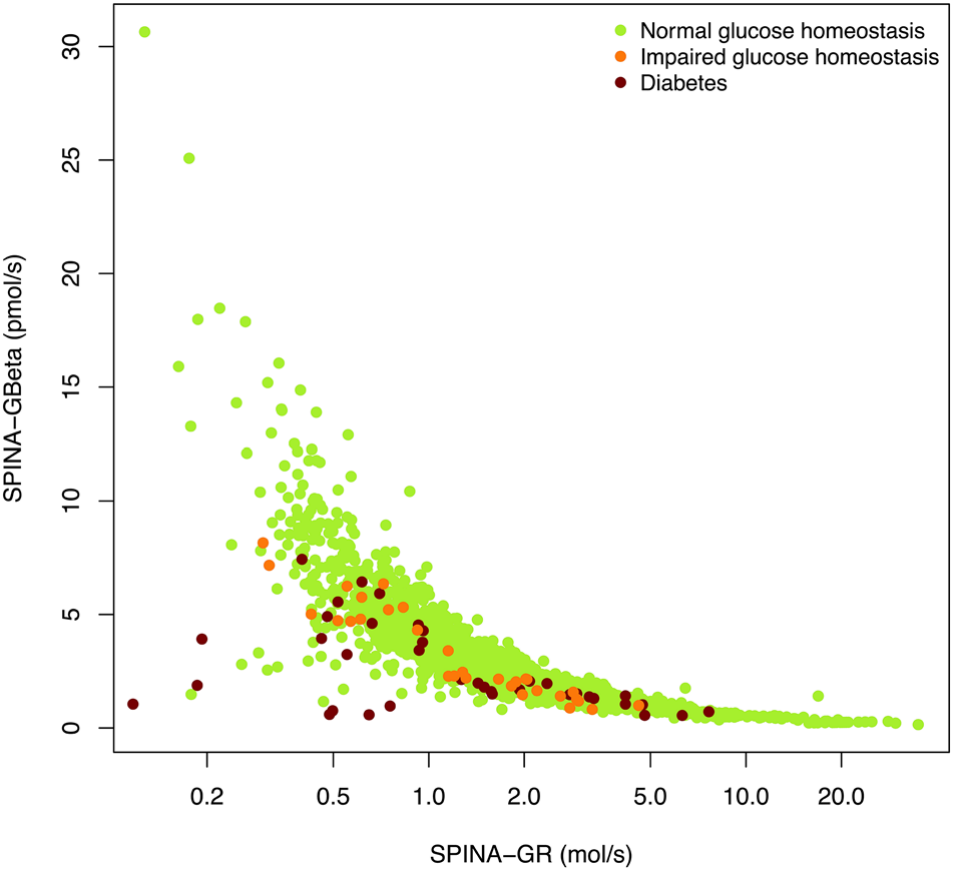
Plot of SPINA-GBeta against SPINA-GR, suggesting a compensatory rise of beta cell function in the case of declining insulin sensitivity in subjects with normal glucose homeostasis (green dots). This ability of beta cell compensation if partly lost in subjects with prediabetes (orange dots) or diabetes (red dots).

The results of both cohorts show a hyperbolic association between SPINA-GBeta and SPINA-GR in healthy volunteers (Fig. 4). Subjects with impaired glucose homeostasis (prediabetes) and diabetes are largely at the lower left edge of this hyperbolic region, indicating comparatively lower functional capacity of beta cells and/or insulin sensitivity.

## Discussion

In this project, we have developed a novel mathematical model of insulin-glucose homeostasis that is founded in basic physiological and biochemical properties of the involved control motifs. Compared with the relatively more complex theories, e. g. the approaches by Sorensen, Misgeld et al. or Panunzi et al.^14,15,41^, it is a relatively simple model that synopses from several details and special interactions. On the other hand, key functional relationships including receptor binding and pharmacokinetic properties are described based on physiological evidence. The design of this model is in direct concordance with the goals of the project, i.e., to develop an analytical solution of steady-state behaviour, to support vertical translation between molecular and whole-organism scales and to lay the foundations of cost-effective and robust diagnostic methods, by simply utilizing fasting insulin and glucose concentrations. On the basis of the MiMe-NoCoDI platform, it was possible to calibrate the model with physiological data from cohort-based studies with a gold standard technique (the hyperinsulinemic euglycaemic clamp study). Under these premises, the fact that simulated concentrations for insulin and glucose were in the normal physiological range supports the assumption of the new model as a sufficient description for glucose homeostasis.

From the equations, we have derived methods to calculate constant structural parameters of the feedback loop, i.e. the secretory capacity of beta cells (*G*_*β*_ or SPINA-GBeta) and the insulin receptor gain (*G*_*R*_ or SPINA-GR), and we validated these parameters in two different ethnic cohorts from two continents, one representing a group of lean young insulin-sensitive men from India and the other one (US citizens from the NHANES 2007/2008 study) being a population-based sample that included older and obese subjects. In both cohorts, SPINA-GBeta and SPINA-GR correlated to key markers of body composition including waist circumference, waist to hip ratio, fat distribution and fat content. More importantly, they also correlated with the results of oral glucose tolerance testing, HbA1c concentration and the glucose disposal rate in a glucose clamp investigation. Additionally, SPINA-GR was significantly reduced in subjects with both diabetes and prediabetes from the NHANES study.

When analysing the fit between traditional and novel parameters, some spurious correlations are to be expected, since HOMA, QUICKI and SPINA employ fasting concentrations of insulin and glucose. The Bland–Altman plots show, however, systematic differences, proving that the approaches do not provide the same information. Therefore, the new static function tests cannot be replaced by the established ones (supplementary Figs. S5–S7).

SPINA-GBeta is similar in the three subgroups of the NHANES cohort (Table 4). This observation may reflect the heterogenous nature of type 2 diabetes comprising subjects with low (SIDD and MARD subtypes) and normal or even compensatory enhanced beta cell function (SIRD and MOD subtypes)^42^. This assumption is supported by the observation that SPINA-GBeta correlates positively to BMI, waist circumference and body fat content and negatively to the *M* value in the clamp investigation.

Plotting SPINA-GBeta against SPINA-GR shows that most subjects with normal glucose homeostasis are within a hyperbolic region, where increased beta cell function compensates for declining insulin sensitivity (Fig. 4). This compensatory capacity may reflect dynamic compensation, where the proliferation of beta cells is stimulated by increased demand^43^. It declines, however, in prediabetes and even more in some subjects with diabetes, which have in part considerably reduced beta-cell function and/or insulin sensitivity. However, the structural parameters of some of the volunteers classified as healthy in the NHANES cohort were located external to the region of physiological compensation as well. Their parameters may indicate a grey zone of undiagnosed prediabetes or even diabetes, where glucose toxicity or the proliferation of senescent cells annihilate the process of dynamic compensation^44^.

The NHANES datasets do not contain any information on a potential allocation of diabetic subjects to the recently defined novel subtypes of diabetes^42^. From theoretical considerations we expect the SAID and SIDD subtypes to be located in the lowest region of Fig. 4 (representing very low SPINA-GBeta), SIRD in the left part of the plot (characterised by low SPINA-GR) and MOD in the lower left region below and/or left of the hyperbolic normal cohort (marked by a combination of comparatively low SPINA-GBeta and SPINA-GR). These assumptions should be confirmed by a dedicated analysis including diabetic subjects with pre-defined modern diabetes classification, however.

In the cohort undergoing the hyperinsulinaemic euglycaemic clamp study that included healthy, lean, young men, the correlations of the novel biomarkers were akin to that of established parameters (HOMA-Beta, HOMA-IR and QUICKI). In the NHANES cohort with its higher proportion of older and obese subjects, however, the correlations were considerably stronger with the novel biomarker panel. This applies particularly to SPINA-GBeta, which shows a significantly stronger correlation with all markers of glucose homeostasis when compared to the corresponding HOMA-Beta parameter. SPINA-GBeta also correlates better with most markers of body composition, with the exception of the triceps skinfold. This advantage of the application in subjects with insulin-resistance reflects the nonlinear nature of the equations used in the new model, which as a result accounts more appropriately for the saturation effects in the context of higher glucose and/or insulin concentrations. Results of longitudinal measurements suggest that SPINA-GBeta, which demonstrates both higher intraindividual *rho* and repeatability (*e*) compared to HOMA-Beta, may be a more reliable biomarker for insulin-glucose homeostasis when compared with traditional parameters. The situation is less clear for HOMA-IR, which displays a lower *e* and higher Spearman’s *rho* when compared with the traditional parameters. In general, the low ergodicity of the calculated parameters indicates that they may represent personalised markers of the metabolic programming in individuals rather than an overall metabolic pattern of the whole cohort. This implies a high degree of intraindividual clustering in insulin-glucose homeostasis, similarly to other endocrine feedback loops, e.g., thyroid homeostasis^45^.

Another advance of the new approach is that it circumvents the “HOMA-blind” zone^46^. Owing to the calculation formula HOMA-Beta cannot be sensibly determined if the fasting glucose concentration is equal or less than 3.5 mmol/l (63 mg/dl). In the situation of a high insulin level combined with a low glucose concentration HOMA-Beta would theoretically get a negative value, although the secretory capacity of beta cells is high in this situation (e.g. in insulinoma or nesidioblastosis). In contrast to HOMA-Beta, the equation for SPINA-GBeta is not affected by this disadvantage and can be calculated over the whole range of possible values for insulin and glucose concentration.

Altogether, mathematical modelling based on non-linear physiological interactions provides novel insights into the physiology of insulin-glucose homeostasis. Additionally, it lays the foundation for the development of novel calculated parameters covering beta-cell function and insulin sensitivity with a higher physiological validity than traditional parameters of the HOMA family. If confirmed by subsequent studies, the novel parameters may be of value for future metabolic research and for screening purposes as well. This may be especially important in the context of ongoing discussions in relation to the utility of calculated parameters of carbohydrate metabolism for purposes of precision diabetes diagnostics and personalised treatments^47,48^.

Future investigations might also evaluate the novel parameters in pregnancy (including gestational diabetes mellitus), ageing and chronic illness. From theoretical reflections a combination of reduced SPINA-GBeta and SPINA-GR may be expected in the senium and chronic illness marked by type 1 allostatic load, whereas reduced SPINA-GR may be compensated by increased SPINA-GBeta in type 2 allostatic load and normal pregnancy. In gestational diabetes we expect some forms of allostatic failure, marked by significantly reduced SPINA-GR and not (or only insufficiently) elevated SPINA-GBeta. These hypotheses have to be validated in carefully planned future studies.

### Limitations

Important modifiers of insulin production and glucose metabolism, e. g. the effects of incretins, glucagon, somatostatin, glucocorticoids, thyroid hormones, cytokines, adipokines, gut microbiota and the autonomic nervous system^49–51^ have not been included in our model. Consequently, the current model will not be able to decipher the impact of these modifiers on the metabolic pattern of an individual. However, the modular structure of the model holds the potential to include additional metabolic modifiers, if required.

Another limitation is the small sample size and low mean BMI of the subjects included in the clamp cohort. The resulting low covariance may be the main reason as to why the correlations of all calculated parameters with the *M* value are rather weak. Therefore, additional validation studies are needed, not only to reassess the findings reported here but to obtain an idea as to whether these results apply to other ethnic populations as well. Generally, it is to be expected that most correlations may be stronger in more heterogeneous ethnic cohorts.

## Conclusions

In summary, our modelling approach represents a compromise between simplicity and physiological fidelity. It tries to implement a balance point between simple descriptions of the insulin-glucose feedback control covered by the homeostasis model assessment and complex models which include those by Sorensen and others. Herein we provide the theoretical basis for a simple and inexpensive static function test based on single fasting insulin and glucose determinations, which has the potential to supplement the currently available spectrum of tests. If further validated, our model might especially be of value in the growing number of adults affected worldwide by the metabolic syndrome and type 2 allostatic load^52^.

## Supplementary Information

Supplementary Information 1.

Supplementary Information 2.

Supplementary Information 3.

## Data Availability

Original data from the NHANES 2007/2008 cohort can be obtained from https://www.cdc.gov/nchs/nhanes/index.htm. The datasets used and analysed from the glucose clamp study are available in the online supplement to this publication. Likewise, S scripts used for evaluations have been provided in the supplementary material to this article. The simulation software SimulaBeta is available along with source code and ready-to-use applications for macOS and Windows from https://simulabeta.sourceforge.io/ or via Zenodo from https://doi.org/10.5281/zenodo.4922800.

## Abbreviations

ASIA: Analog Signal memory with Intrinsic Adjustment
BMI: Body mass index
CV: Coefficient of variation
DXA: Dual Energy X-ray absorptiometry
FFM: Fat-free mass
fsIGT: Frequently sampled intravenous glucose tolerance test
GBeta or G_β_: Gain of beta-cells
GR: Insulin receptor gain
HbA1c: Glycohemoglobin
HEC: Hyperinsulinemic-euglycemic clamp
HOMA: Homeostasis model assessment
IDE: Integrated development environment
IR: Insulin resistance
IRB: Institutional review board
MARD: Mild age-related diabetes
MOD: Mild obesity-related diabetes
MOE: Ministry of education
MiMe: Michaelis–Menten
NHANES: National health and nutrition examination survey
NoCoDI: Non-competitive divisive inhibition
OGTT: Oral glucose tolerance testing
ORDAC: Overrange detection and correction
QUICKI: Quantitative insulin sensitivity check index
ROI: Region of interest
SAID: Severe autoimmune diabetes
SIDD: Severe insulin-deficient diabetes
SIRD: Sever insulin-resistant diabetes
SPINA: Structure parameter inference approach
V_D_: Volume of distribution
