# Supplementary material for: SPINA Carb: a simple mathematical model supporting fast in-vivo estimation of insulin sensitivity and beta cell function
Source: Sci Rep. 2022 Oct 21;12:17659. doi: 10.1038/s41598-022-22531-3 (PMC9587026; doi:10.1038/s41598-022-22531-3)
Supplement: Supplementary file 3 — Supplementary Information 3. [file 41598_2022_22531_MOESM3_ESM.pdf]

Supplementary Material Accompanying the Publication "SPINA Carb: A simple mathematical model supporting fast in-vivo estimation of insulin sensitivity and beta cell function"

Johannes W. Dietrich*, Riddhi Dasgupta, Shajith Anoop, Felix Jebasingh, Mathews E Kurian, Mercy Inbakumari, Bernhard O. Boehm, Nihal Thomas

***Correspondence:** Corresponding Author: johannes.dietrich@ruhr-uni-bochum.de

1 Comprehensive Mathematical Exposition and Dimensional Analysis

In order to clarify the relationship between the elements of the model, units of measurement are reported in their base form without multipliers / prefixes, e. g. mol/L instead or mmol/L.

ASIA element for glucose:

$$G_1 = \frac{\alpha_G}{\beta_G} \qquad \text{s/L} \qquad (1)$$

Glucose concentration:

$$G(t) = G_1 R(t) \qquad \text{mol/L} \qquad (2)$$

Insulin secretion rate:

$$S(t) = \frac{G_\beta G(t)}{D_\beta + G(t)} \qquad \text{mol/s} \qquad (3)$$

ASIA element for insulin:

$$G_3 = \frac{\alpha_I}{\beta_I} \qquad \text{s/L} \qquad (4)$$

Insulin concentration:

$$I(t) = G_3 S(t) \qquad \text{mol/L} \qquad (5)$$

Primary insulin signaling:

$$M(t) = \frac{G_R I(t)}{D_R + I(t)} \qquad \text{mol/s} \qquad (6)$$

Secondary insulin signaling:

$$N(t) = G_E M(t) \qquad 1 \qquad (7)$$

Regulated glucose production rate:

$$Q(t) = \frac{P(t)}{1 + N(t)} \qquad \text{mol/s} \qquad (8)$$

Glucose arrival rate:

$$R(t) = W(t) + Q(t) \qquad \text{mol/s} \qquad (9)$$

Glucose concentration in terms of insulin concentration:

$$G(t) = G_1 W(t) + \frac{G_1 P(t)}{1+\frac{G_E G_R [I](t)}{D_R + [I](t)}} \qquad \text{mol/L} \qquad (10)$$

Insulin concentration in terms of glucose concentration:

$$I(t) = \frac{G_3 G_\beta G(t)}{D_\beta + G(t)} \qquad \text{mol/L} \qquad (11)$$

Iterative equation for glucose concentration:

$$G(t+1) = G_1 W(t) + \frac{G_1 P(t)}{1+\frac{K_1 G(t)}{K_2 + G(t)}} \qquad \text{mol/L} \qquad (12)$$

with

$$K_1 = \frac{G_E G_R G_3 G_\beta}{D_R + G_3 G_\beta} \qquad 1 \qquad (13)$$

and

$$K_2 = \frac{D_R D_\beta}{D_R + G_3 G_\beta}. \qquad \text{mol/L} \qquad (14)$$

For fasting steady-state situations, given by $W(t) = 0$ and $t \rightarrow \infty$, equation (11) can be rewritten with

$$a = 1 + K_1, \qquad 1 \qquad (15)$$

$$b = K_2 - G_1 P(\infty) \qquad \text{mol/L} \qquad (16)$$

and

$$c = -G_1 K_2 P(\infty) \qquad \text{mol}^2/\text{L}^2 \qquad (17)$$

as the quadratic equation

$$aG(\infty)^2 + bG(\infty) + c = 0. \qquad \text{mol}^2/\text{L}^2 \qquad (18)$$

Since all parameters are positive, the relation $b < b^2 - 4ac > 0$ is necessarily fulfilled. Therefore, equation (17) has the two solutions

$$G(\infty)_{1,2} = \frac{-b \pm \sqrt{b^2 - 4ac}}{2a}. \qquad \text{mol/L} \qquad (19)$$

The positive solution is the only one that is possible in a physiological context. It represents the equifinal glucose concentration in the fasting steady-state.

2 Methods for the determination of P_0

For predicting the steady state and computer simulations, it is necessary to define the constitutive (unregulated) endogenous glucose production rate P_0. This is possible in several ways:

One method is to rearrange equation (8) and solve for P with

$$P = Q + QN. \tag{14}$$

Required parameters are N (which can be derived from expected insulin concentrations) and Q. Under the assumption of fasting steady-state conditions, Q can be obtained from glucose disposal rate in physiological studies (which is necessarily identical to the glucose arrival rate R, since, by definition, $W = 0$ and $\frac{d[G]}{dt} = 0$).

Another approach would be to solve the model equations in steady-state with a defined fasting glucose concentration G_∞ for P. A graphical illustration of this method is shown in supplementary figure 1.

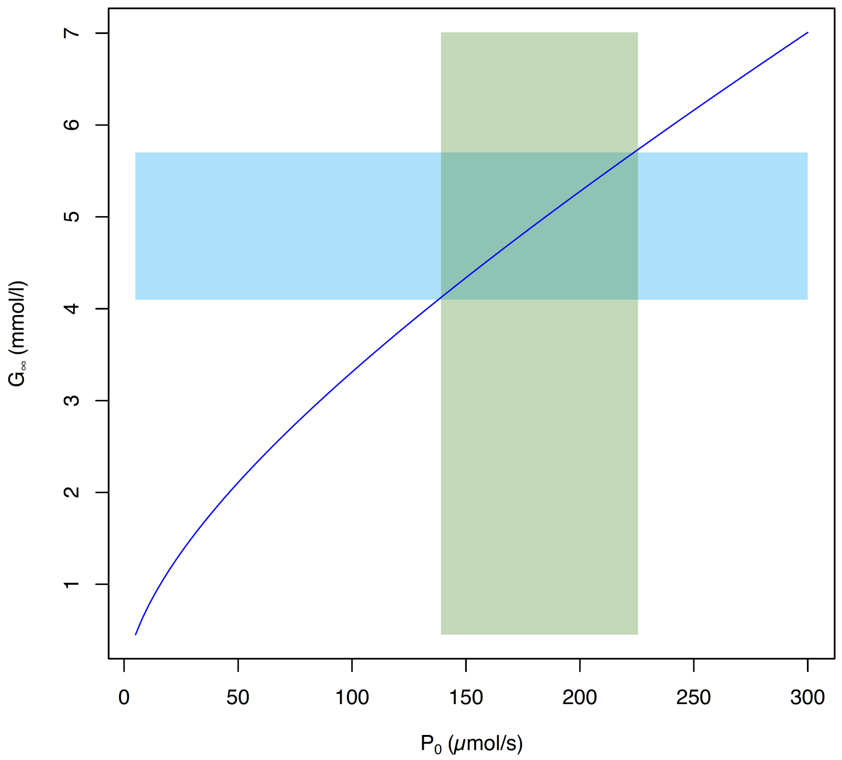

Supplementary Figure 1: Determining the external glucose arrival rate P_0 from fasting steady-state glucose concentrations G_∞. The blue rectangle represents the reference range for fasting glucose (here taken from the glucose clamp cohort of this analysis). Then the green rectangle delivers the resulting range for normal P_0 values.

3 Detailed results of computer simulation

Supplementary Table 1: *Fasting steady state values as predicted by the model, compared to established laboratory reference ranges*

Variable	Explanation	Predicted value	Reference range
Q	Regulated endogenous glucose production rate	28.0 µmol/s	N/A
R	Glucose arrival rate	28.0 µmol/s	N/A
$[G]$	Glucose concentration	4.3 mmol/L	3.3–5.6 mmol/L
S	Insulin secretion rate	1.1 pmol/s	N/A
$[I]$	Insulin concentration	63.0 pmol/L	< 138 pmol/L
M	Primary insulin signalling	0.09 nmol/s	N/A

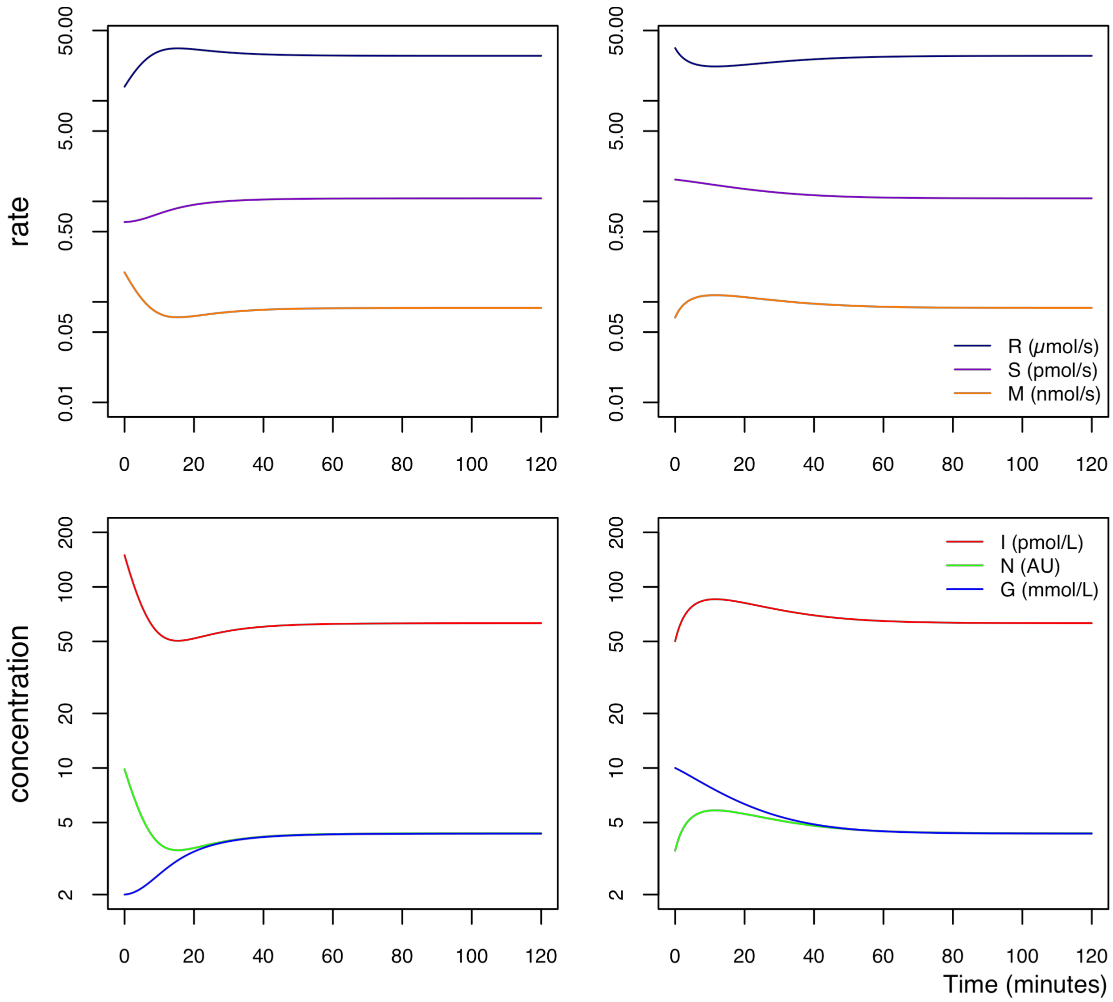

Supplementary Figure 2: *Results of computer simulations over 120 minutes. Initial conditions for left panels: P_0 = 150 µmol/s, $[I]_0$ = 150 pmol/l, $[G]_0$ = 2 mmol/l. Right panels: P_0 = 150 µmol/s, $[I]_0$ = 50 pmol/l, $[G]_0$ = 10 mmol/l.*

Supplementary Table 2: *Results of simulated dynamic testing*

**Parameter**	**Explanation**	**Simulated result**	**Reference range**
Oral glucose tolerance test (oGTT)			
G_{2h}	2h glucose concentration	7.45 mmol/L	< 7.77 mmol/L
I_{2h}	2h insulin concentration	86.1 pmol/L	< 207 pmol/L
Frequently sampled intravenous glucose tolerance test (fsIGT)			
k	Canard's glucose assimilation coefficient	3,15%	> 1.4%

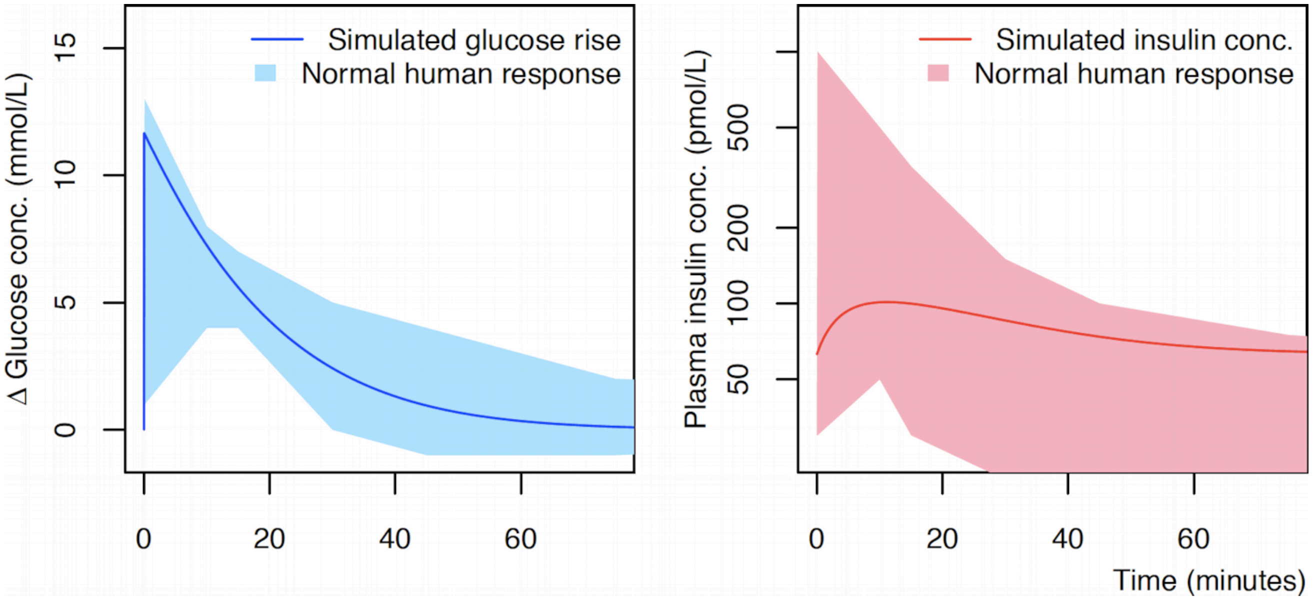

Supplementary Figure 3: *Simulations of a frequently sampled intravenous glucose tolerance test (fsIGT). Shown are the responses of glucose (left panel) and insulin concentration (right panel). The respective shaded areas represent the time-dependent reference ranges for healthy volunteers.*

4 Relation between traditional and novel calculated parameters

The following plots (supplementary figures 4 to 6) show the relationship between traditional (HOMA and QUICKI) and novel parameters (SPINA). Since both approaches are based on fasting concentrations of insulin and glucose, some spurious correlation is to be expected. The Bland-Altman plots show systematic differences, proving that the two approaches don't provide the same information.

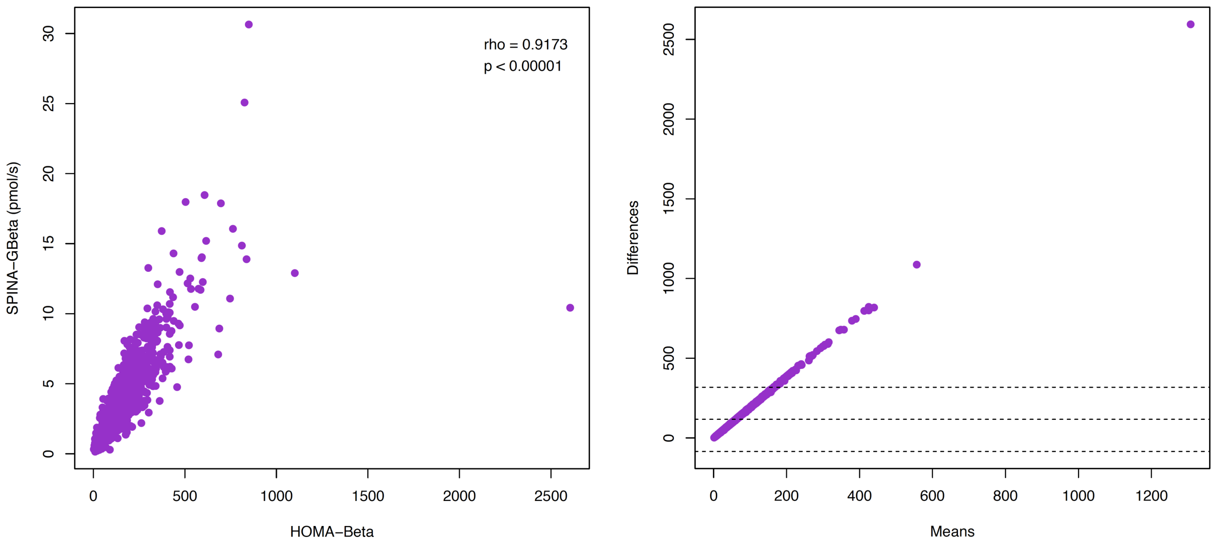

Supplementary Figure 4: Correlation between SPINA-GBeta and HOMA-Beta (left) and Bland-Altman diagram of the two parameters (right)

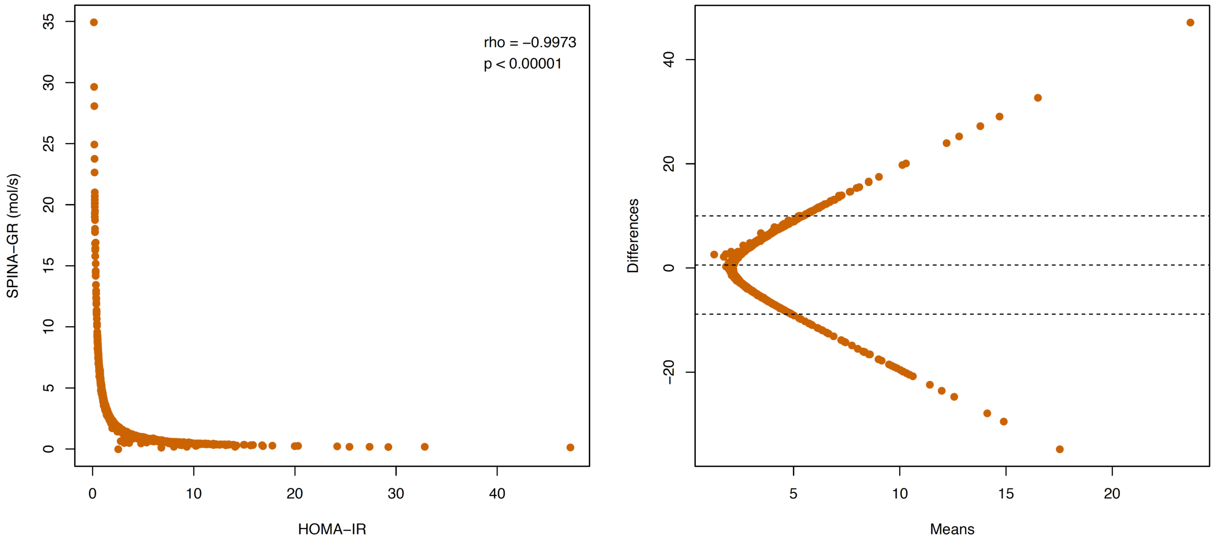

Supplementary Figure 5: Correlation between SPINA-GR and HOMA-IR (left) and Bland-Altman diagram of these parameters (right)

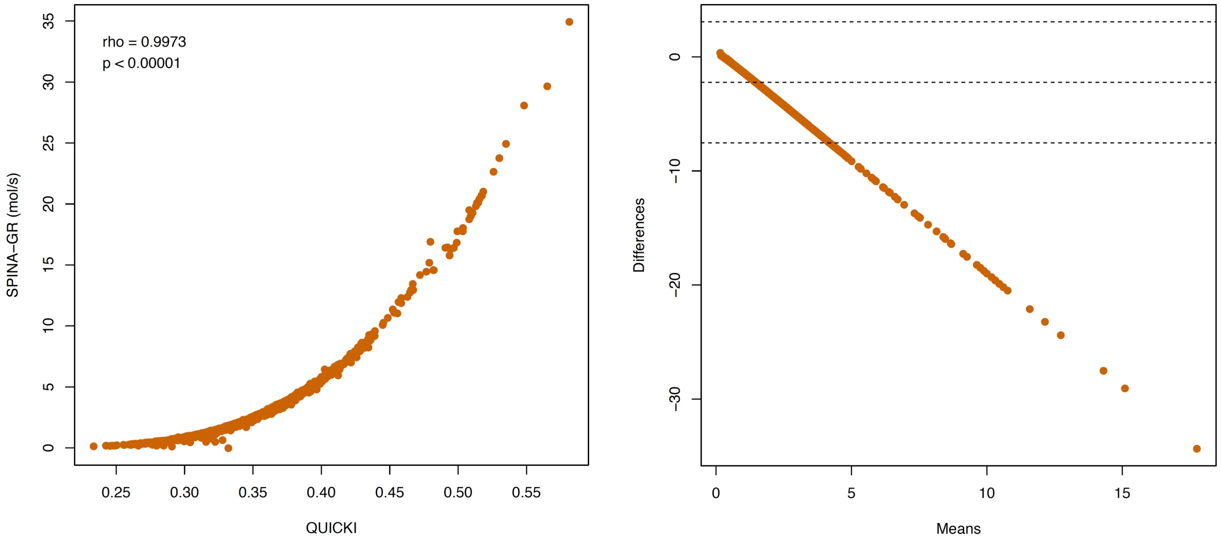

Supplementary Figure 6: Correlation between SPINA-GR and QUICKI (left) and Bland-Altman diagram of the two parameters (right)

5 Ergodicity

Supplementary Table 3: *Test-retest reliability and ergodicity of calculated biomarkers from repeated measurements. See Methods section for calculation of e. Larger Spearman's rho values denote higher reliability and higher e values represent lower ergodicity. ††† p < 1e–15*

**Parameter**	***e***	**Spearman's *rho***
HOMA-Beta	0.79	0.733†††
HOMA-IR	0.87	0.765†††
QUICKI	0.77	0.765†††
SPINA-GBeta	0.85	0.755†††
SPINA-GR	0.66	0.766†††

6 S Functions for calculating SPINA-GBeta and SPINA-GR

The following functions can be used to calculate the novel structure parameters with standard implementations of the statistical language S, e. g. with the R environment 3.5 or newer:

```
SPINA.GBeta <- function(Insulin, Glucose)
# Insulin expected in pmol/l, Glucose in mmol/l
{
  pico.factor <- 1e12;
  mili.factor <- 1e3
  betaI <- 3.4e-3;
  alphaI <- 0.2;
  dBeta <- 7e-3;
  GBeta <- pico.factor * betaI * Insulin / pico.factor * (dBeta + Glucose /
    mili.factor) / (alphaI * Glucose / mili.factor);
  return(GBeta);
}

SPINA.GR <- function(Insulin, Glucose)
# Insulin in pmol/l, Glucose in mmol/l
{
  pico.factor <- 1e12;
  mili.factor <- 1e3;
  alphaG <- 0.11;
  betaG <- 7.1e-4;
  P0 <- 150e-6;
  DR <- 1.6e-9;
  GE <- 50;
  GR <- alphaG * P0 * (DR + Insulin / pico.factor) / (betaG * GE * Insulin /
    pico.factor * Glucose / mili.factor) - DR / (GE * Insulin / pico.factor)
    - 1 / GE;
  return(GR);
}
```

7 Source code for the simulation unit of SimulaBeta

The source code of the simulation unit is shown for illustration and explanation only. This code requires Free Pascal (version 3.0 or newer), Lazarus (version 2.0 or newer), and the CyberUnits Bricks Library to be compiled. Compiling requires additional units for the user interface and services that are part of the SimulaBeta project. SimulaBeta is available online along with source code and a precompiled executable application for macOS and Windows from https://sourceforge.net/projects/simulabeta/ or *via* https://doi.org/10.5281/zenodo.4922800.

```
unit SimulationEngine;

{ SimulaBeta }
{ A simulator for insulin-glucose homeostasis }
{ Simulation Engine }

{ Version 2.2.0 (Malakow) }

{ (c) Johannes W. Dietrich, 1994 - 2022 }
{ (c) Ludwig Maximilian University of Munich 1995 - 2002 }
{ (c) University of Ulm Hospitals 2002-2004 }
{ (c) Ruhr University of Bochum 2005 - 2022 }

{ Source code released under the BSD License }

{ See the file "license.txt", included in this distribution, }
{ for details about the copyright. }
{ Current versions and additional information are available from }
{ http://simulabeta.sf.net }

{ This program is distributed in the hope that it will be useful, }
{ but WITHOUT ANY WARRANTY; without even the implied warranty of }
{ MERCHANTABILITY or FITNESS FOR A PARTICULAR PURPOSE. }

{$mode objfpc}{$H+}

interface

uses
  Classes, SysUtils, Forms, bricks, lifeblocks;
```

```
type
  tTestKind = (tkNone, tkfsIGT, tkoGTT);
  tParameterSpace = record
    alphaG, betaG, alphaI, betaI, GBeta, DBeta, GR, DR, GE: extended;
  end;
  tUnits = record
    P, Q, R, G, S, I, M, N, W: string;
  end;
  tTestInfo = record
    kind: tTestKind;
    startTime: integer;
  end;


const
  MilliFactor = 1e-3;
  MicroFactor = 1e-6;
  NanoFactor = 1e-9;
  PicoFactor = 1e-12;

  MGlucose = 180.156;  // molar mass in g/mol

  {Conversions:
  alpha = 1 / VD
  t1/2 = VD * ln2 / Clearance
  beta = ln2 / t1/2 = Clearance / VD
  }

  InitialStrucPars: tParameterSpace =
  (
  alphaG: 0.11;          // 1 / VD for glucose (initial phase, 1/L)
                         // [Hirota et al. 1999, PMID 10233198]: 7.24 and 7.27 L
                         // [Sjoestrand et al. 2001, PMID 11878683: 12.3 L
                         // [Sjoestrand and Hahn 2004, PMID 14977794]: 9.14 L
                         // [van Tulder et al. 2005, PMID 16192526]: 90 mL/kg
  betaG: 7.1e-4;         // Clearance exponent (ln2 / half-life) for glucose
                         // [Sjoestrand et al. 2001, PMID 11878683]: t1/2 12.1 min
                         // [Sjoestrand and Hahn 2004, PMID 14977794]: t1/2 11..16 min
                         // [Strandberg and Hahn 2005, PMID 15486008]: t1/2 12..30 min
  alphaI: 0.2;           // 1 / VD for insulin (1/l)
                         // [Rang, H. P. (2003). Pharmacology. Edinburgh:
```

```
                          //    Churchill Livingstone. ISBN 0-443-07145-4]
  betaI: 3.4e-3;          // Clearance exponent (Clearance / VD) for insulin
                          // [Turnheim and Waldhaeusl 1988, PMID 3281377]]: 700..800 mL/min
                          // [[Weiss et al. 2015, PMID 26608654]: CL 141..571 mL/min
                          // [[Koschorreck and Gilles 2008, PMID 18477391]: 700..3350 mL/min
  GBeta: 2.8e-12;         // Estimated from NHANES study (mol/s)
  DBeta: 7e-3;            // EC50 of glucose (mol/l)
                          // [Byrne et al. 1994, PMID 8132752]
                          // [Jones et al. 1997, PMID 9177392]: ca. 7 mmol/L
                          // [Jones et al. 2000, PMID 10710505]: ca. 6 mmol/L
                          // Toschi et al. 2002, PMID 11815471]: ca 8 mmol/L
  GR: 2.3;                // Estimated from NHANES study
  DR: 1.6e-9;             // EC50 of insulin (mol/l)
                          // [Natali et al. 2000, PMID 10780934]: 240 mU/L
  GE: 50;                 // Calibration factor
  );

  PFactor = MicroFactor;
  P0 = 150 * PFactor;     // Glucose arrival (production + absorption)
                          // Estimated to deliver fasting R between 10 and
                          // 100 mcmol/s
                          // R = P / (1 + N)  =>  P = R + RN
                          // [Sjoestrand and Hahn 2004, PMID 14977794]: 15..85 mcmol/s
                          // [Giebelstein et al. 2012, PMID 22282162]: 129 mcmol/s
  IFactor = PicoFactor;
  I0 = 100 * IFactor;     // Fasting insulin concentration in mol/l
                          // should be 20 to 210 pmol/l (3 to 30 mIU/l)
                          // [Giebelstein et al. 2012, PMID 22282162]: 24 pmol/l
  GFactor = MilliFactor;
  G0 = 5 * GFactor;       // Fasting glucose concentration in mol/l
                          // should be 3.3 to 5.5 mmol/l (60 to 100 mg/dl)
  WFactor = MicroFactor;

type

  { TValues }

  TValues = class
  protected
    function GetSize: integer;
    procedure SetSize(aValue: integer);
  public
```

```
    t, P, Q, R, G, S, I, M, N, W: array of extended;
    constructor Create;
    destructor Destroy;
    property size: integer read GetSize write SetSize;
  end;

  TBlocks = record
    G1, G3: TASIA;
    MiMeBeta, MiMeR: TMiMe;
    GE: TP;
    NoCoDI: TNoCoDI;
  end;

  TSolution = record
    P, W, Q, R, G, S, I, M, N: extended;
  end;
  TPrediction = array[0..1] of TSolution;
  TQRoots = array[0..1] of extended;

var
  gStrucPars: tParameterSpace;
  gValues: TValues;
  gUnits: TUnits;
  t: extended;
  delta: real;
  gTestInfo: tTestInfo;

procedure InitUnits;
procedure InitSimulation;
function PredictedEquilibrium(P: extended; StrucPars: tParameterSpace): TPrediction;
procedure RunSimulation(P, Glc, Ins: extended; nmin, nmax: integer; prediction: TPrediction);

implementation

function SolveQuadratic(a, b, c: extended): TQRoots;
{ solves quadratic equation with parameters a, b and c }
begin
  Result[0] := -(b + sqrt(sqr(b) - 4 * a * c)) / (2 * a);
  Result[1] := -(b - sqrt(sqr(b) - 4 * a * c)) / (2 * a);
end;
```

```
procedure InitUnits;
begin
  gUnits.G := 'mmol/L';
  gUnits.I := 'pmol/L';
  gUnits.P := 'µmol/L';
  gUnits.Q := 'µmol/s';
  gUnits.R := 'µmol/s';
  gUnits.S := 'pmol/s';
  gUnits.M := 'nmol/s';
  gUnits.N := '';
  gUnits.W := 'µmol/s';
end;

procedure InitSimulation;
begin
  if assigned(gValues) then
    gValues.Size := 0 // delete content
  else
    gValues := TValues.Create;
  gStrucPars := InitialStrucPars;
  delta := 1;
  t := 0;
  gTestInfo.kind := tkNone;
  gTestInfo.startTime := 0;
end;

procedure SetInitialConditions(Prediction: TPrediction);
{ sets initial conditions from predicted equilibrium, modifiable by GUI }
var
  i: 0..1;
begin
  if gValues.size > 0 then
  begin
    if Prediction[0].G < 0 then  // use positive solution for initial values
      i := 1
    else
      i := 0;
    gValues.t[0] := 0;
    gValues.P[0] := Prediction[i].P;
    gValues.R[0] := Prediction[i].R;
    gValues.G[0] := Prediction[i].G;
    gValues.S[0] := Prediction[i].S;
```

```
    gValues.I[0] := Prediction[i].I;
    gValues.M[0] := Prediction[i].M;
    gValues.N[0] := Prediction[i].N;
    gValues.W[0] := 0;;
  end;
end;

function PredictedEquilibrium(P: extended; StrucPars: tParameterSpace): TPrediction;
var
    a, b, c, K1, K2: extended;
    G1, G3: extended;
begin
  G1 := StrucPars.alphaG / StrucPars.betaG; // Gain of ASIA element
  G3 := StrucPars.alphaI / StrucPars.betaI; // Gain of ASIA element
  Result[0].P := P;
  Result[1].P := P;
  Result[0].W := 0;
  Result[1].W := 0;

  { Solving for G: }
  with StrucPars do
  begin
    K1 := GE * GR * G3 * GBeta / (DR + G3 * GBeta);
    K2 := DR * DBeta / (DR + G3 * GBeta);

    a := 1 + K1;
    b := K2 - G1 * Result[0].P;
    c := -G1 * K2 * Result[1].P;

    Result[0].G := SolveQuadratic(a, b, c)[0];
    Result[0].S := GBeta * Result[0].G / (DBeta + Result[0].G);
    Result[0].I := G3 * Result[0].S;
    Result[0].M := GR * Result[0].I / (DR + Result[0].I);
    Result[0].N := GE * Result[0].M;
    Result[0].Q := Result[0].P / (1 + Result[0].N);
    Result[0].R := Result[0].Q;

    Result[1].G := SolveQuadratic(a, b, c)[1];
    Result[1].S := GBeta * Result[1].G / (DBeta + Result[1].G);
    Result[1].I := G3 * Result[1].S;
    Result[1].M := GR * Result[1].I / (DR + Result[1].I);
```

```
    Result[1].N := GE * Result[1].M;
    Result[1].Q := Result[1].P / (1 + Result[1].N);
    Result[1].R := Result[1].Q;
  end;
end;

procedure RunSimulation(P, Glc, Ins: extended; nmin, nmax: integer; prediction: TPrediction);
const
  w0 = 0.5;                          // Calibrated
  p1 = 1 / w0 - 1;                   // derived from Subba Rao et al. 1990 and Lenbury et al. 2001
  betaGI = ln(2) / (50 * SecsPerMin);  // based on half-life of intestinal glucose
                                     // [Dalla Man et al. 2004 and Anderwald et al. 2010]
  f0 = 0.85;                         // bio-availability of glucose
                                     // [Dalla Man et al. 2004 and Anderwald et al. 2010]
  c0 = 68e-3 / (3e-3 / SecsPerMin);    // Calibration factor gut glucose absorption
                                     // [Brubaker et al 2007]: ca. 3 mmol/min
var
  blocks: TBlocks;
  Q, R, S, M, N, W: extended;
  i: integer;
begin
  W := 0;
  if nmax > 0 then
  begin
    blocks.G1 := TASIA.Create;
    blocks.G3 := TASIA.Create;
    blocks.GE := TP.Create;
    blocks.MiMeBeta := TMiMe.Create;
    blocks.MiMeR := TMiMe.Create;
    blocks.NoCoDI := TNoCoDI.Create;

    with gStrucPars do
    begin
      blocks.G1.alpha := alphaG;
      blocks.G1.beta := betaG;
      blocks.G1.delta := delta;
      blocks.MiMeBeta.G := GBeta;
      blocks.MiMeBeta.D := DBeta;
      blocks.G3.alpha := alphaI;
      blocks.G3.beta := betaI;
      blocks.G3.delta := delta;
      blocks.MiMeR.G := GR;
```

```
    blocks.MiMeR.D := DR;
    blocks.GE.G := GE;
    // SetInitialConditions(prediction);  // for future extension
    N := GE * GR * Ins / (DR + Ins);
  end;
  blocks.G1.x1 := Glc; // "prefill" memory elements...
  blocks.G3.x1 := Ins; // ...with provided values
  for i := nmin to nmax do
  begin
    blocks.NoCoDI.input1 := P;
    blocks.NoCoDI.input2 := N;
    Q := blocks.NoCoDI.simOutput;
    if gTestInfo.kind = tkOGTT then
      begin
        if i >= gTestInfo.startTime then
        begin
          // Dosage 75 g
          // [Subba Rao et al. 1990 and Lenbury et al. 2001]
          W := 75 / MGlucose * f0 / c0 / (p1 + exp(betaGI * t));
        end
      end;
    R := Q + W;
    blocks.G1.input := R;
    Glc := blocks.G1.simOutput;
    if gTestInfo.kind = tkfsIGT then
      begin
        if i >= gTestInfo.startTime then
        begin
          // Dosage 21 g for 70 kg
          Glc := Glc + (gStrucPars.alphaG * 21 / MGlucose);
          blocks.G1.x1 := Glc;
          gTestInfo.kind := tkNone; // switch off again
        end;
      end;
    blocks.MiMeBeta.input := Glc;
    S := blocks.MiMeBeta.simOutput;
    blocks.G3.input := S;
    Ins := blocks.G3.simOutput;
    blocks.MiMeR.input := Ins;
    M := blocks.MiMeR.simOutput;
    blocks.GE.input := M;
```

```
      N := blocks.GE.simOutput;
      t := t + delta;
      gValues.P[i] := P;
      gValues.Q[i] := Q;
      gValues.R[i] := R;
      gValues.G[i] := Glc;
      gValues.S[i] := S;
      gValues.I[i] := Ins;
      gValues.M[i] := M;
      gValues.N[i] := N;
      gValues.W[i] := W;
      gValues.t[i] := t;
      application.ProcessMessages;
    end;
    blocks.G1.Destroy;
    blocks.G3.Destroy;
    blocks.MiMeBeta.Destroy;
    blocks.MiMeR.Destroy;
    blocks.GE.Destroy;
    blocks.NoCoDI.Destroy;
  end;
end;

{ TValues }

function TValues.GetSize: integer;
begin
  result := Length(R);
end;

procedure TValues.SetSize(aValue: integer);
begin
  SetLength(t, aValue);
  SetLength(P, aValue);
  SetLength(Q, aValue);
  SetLength(R, aValue);
  SetLength(G, aValue);
  SetLength(S, aValue);
  SetLength(I, aValue);
  SetLength(M, aValue);
  SetLength(N, aValue);
  SetLength(W, aValue);
```

```
end;

constructor TValues.Create;
begin
  inherited Create;
  Size := 0;
end;

destructor TValues.Destroy;
begin
  inherited Destroy;
end;

end.

{ References:
```

Hirota K, Ishihara H, Tsubo T, Matsuki A. Estimation of the initial distribution volume of glucose by an incremental plasma glucose level at 3 min after i.v. glucose in humans. Br J Clin Pharmacol. 1999 Apr;47(4):361-4. doi: 10.1046/j.1365-2125.1999.00889.x. PMID: 10233198; PMCID: PMC2014248.

Sjöstrand F, Edsberg L, Hahn RG. Volume kinetics of glucose solutions given by intravenous infusion. Br J Anaesth. 2001 Dec;87(6):834-43. doi: 10.1093/bja/87.6.834. Erratum in: Br J Anaesth 2002 May;88(5):753. PMID: 11878683.

Sjöstrand F, Hahn RG. Volume kinetics of glucose 2.5% solution during laparoscopic cholecystectomy. Br J Anaesth. 2004 Apr;92(4):485-92. doi: 10.1093/bja/aeh095. Epub 2004 Feb 20. PMID: 14977794.

van Tulder L, Michaeli B, Chioléro R, Berger MM, Revelly JP. An evaluation of the initial distribution volume of glucose to assess plasma volume during a fluid challenge. Anesth Analg. 2005 Oct;101(4):1089-1093. doi: 10.1213/01.ane.0000167769.84459.b7. PMID: 16192526.

Strandberg P, Hahn RG. Volume kinetics of glucose 2.5% solution and insulin resistance after abdominal hysterectomy. Br J Anaesth. 2005 Jan;94(1):30-8. doi: 10.1093/bja/aeh285. Epub 2004 Oct 14. PMID: 15486008.

Rang, H. P. (2003). Pharmacology. Edinburgh: Churchill Livingstone.

ISBN 0-443-07145-4

Turnheim K, Waldhäusl WK. Essentials of insulin pharmacokinetics. Wien Klin Wochenschr. 1988 Feb 5;100(3):65-72. PMID: 3281377.

Weiss M, Tura A, Kautzky-Willer A, Pacini G, D'Argenio DZ. Human insulin dynamics in women: a physiologically based model. Am J Physiol Regul Integr Comp Physiol. 2016 Feb 1;310(3):R268-74. doi: 10.1152/ajpregu.00113.2015. Epub 2015 Nov 25. PMID: 26608654; PMCID: PMC4796751.

Koschorreck M, Gilles ED. Mathematical modeling and analysis of insulin clearance in vivo. BMC Syst Biol. 2008 May 13;2:43. doi: 10.1186/1752-0509-2-43. PMID: 18477391; PMCID: PMC2430945.

Byrne MM, Sturis J, Clément K, Vionnet N, Pueyo ME, Stoffel M, Takeda J, Passa P, Cohen D, Bell GI, et al. Insulin secretory abnormalities in subjects with hyperglycemia due to glucokinase mutations. J Clin Invest. 1994 Mar;93(3):1120-30. doi: 10.1172/JCI117064. PMID: 8132752; PMCID: PMC294056.

Jones CN, Pei D, Staris P, Polonsky KS, Chen YD, Reaven GM. Alterations in the glucose-stimulated insulin secretory dose-response curve and in insulin clearance in nondiabetic insulin-resistant individuals. J Clin Endocrinol Metab. 1997 Jun;82(6):1834-8. doi: 10.1210/jcem.82.6.3979. PMID: 9177392.

Jones CN, Abbasi F, Carantoni M, Polonsky KS, Reaven GM. Roles of insulin resistance and obesity in regulation of plasma insulin concentrations. Am J Physiol Endocrinol Metab. 2000 Mar;278(3):E501-8. doi: 10.1152/ajpendo.2000.278.3.E501. PMID: 10710505.

Toschi E, Camastra S, Sironi AM, Masoni A, Gastaldelli A, Mari A, Ferrannini E, Natali A. Effect of acute hyperglycemia on insulin secretion in humans. Diabetes. 2002 Feb;51 Suppl 1:S130-3. doi: 10.2337/diabetes.51.2007.s130. PMID: 11815471.

Natali A, Gastaldelli A, Camastra S, Sironi AM, Toschi E, Masoni A, Ferrannini E, Mari A. Dose-response characteristics of insulin action on glucose metabolism: a non-steady-state approach. Am J Physiol Endocrinol Metab. 2000 May;278(5):E794-801. doi: 10.1152/ajpendo.2000.278.5.E794. PMID: 10780934.

Giebelstein J, Poschmann G, Højlund K, Schechinger W, Dietrich JW, Levin K, Beck-Nielsen H, Podwojski K, Stühler K, Meyer HE, Klein HH. The proteomic

signature of insulin-resistant human skeletal muscle reveals increased glycolytic and decreased mitochondrial enzymes. Diabetologia. 2012 Apr;55(4):1114-27. doi: 10.1007/s00125-012-2456-x. Epub 2012 Jan 27. Erratum in: Diabetologia. 2012 Jul;55(7):2083. PMID: 22282162.

Dalla Man C, Caumo A, Basu R, Rizza R, Toffolo G, Cobelli C. Minimal model estimation of glucose absorption and insulin sensitivity from oral test: validation with a tracer method. Am J Physiol Endocrinol Metab. 2004 Oct;287(4):E637-43. doi: 10.1152/ajpendo.00319.2003. Epub 2004 May 11. PMID: 15138152.

Anderwald C, Gastaldelli A, Tura A, Krebs M, Promintzer-Schifferl M, Kautzky-Willer A, Stadler M, DeFronzo RA, Pacini G, Bischof MG. Mechanism and effects of glucose absorption during an oral glucose tolerance test among females and males. J Clin Endocrinol Metab. 2011 Feb;96(2):515-24. doi: 10.1210/jc.2010-1398. Epub 2010 Dec 8. PMID: 21147888.

Lenbury Y, Ruktamatakul S, Amornsamarnkul S. Modeling insulin kinetics: responses to a single oral glucose administration or ambulatory-fed conditions. Biosystems. 2001 Jan;59(1):15-25. doi: 10.1016/s0303-2647(00)00136-2. PMID: 11226623.

Subba Rao G, Bajaj JS, Subba Rao J. A mathematical model for insulin kinetics. II. Extension of the model to include response to oral glucose administration and application to insulin-dependent diabetes mellitus (IDDM). J Theor Biol. 1990 Feb 22;142(4):473-83. doi: 10.1016/s0022-5193(05)80103-1. PMID: 2187116.

Brubaker PL, Ohayon EL, D'Alessandro LM, Norwich KH. A mathematical model of the oral glucose tolerance test illustrating the effects of the incretins. Ann Biomed Eng. 2007 Jul;35(7):1286-300. doi: 10.1007/s10439-007-9274-1. PMID: 17393338.
}
